# Comparative Long-Term Effectiveness of a Monotherapy with Five Antiepileptic Drugs for Focal Epilepsy in Adult Patients: A Prospective Cohort Study

**DOI:** 10.1371/journal.pone.0131566

**Published:** 2015-07-06

**Authors:** Qing-Yi Zeng, Tian-Tian Fan, Pan Zhu, Ru-Qian He, Yi-Xin Bao, Rong-Yuan Zheng, Hui-Qin Xu

**Affiliations:** 1 Department of Neurology, The First Affiliated Hospital of Wenzhou Medical University, Wenzhou, Zhejiang Province, P.R. China; 2 Rehabilitation Center, The First Hospital of Zibo, Zibo, Shandong Province, P.R. China; The George Washington University, UNITED STATES

## Abstract

**Objective:**

To evaluate and compare long-term effectiveness of five antiepileptic drugs (AEDs) for monotherapy of adult patients with focal epilepsy in routine clinical practice.

**Methods:**

Adult patients with focal epilepsy, who were prescribed with carbamazepine (CBZ), valproate (VPA), lamotrigine (LTG), topiramate (TPM), or oxcarbazepine (OXC) as monotherapy, during the period from January 2004 to June 2012 registered in Wenzhou Epilepsy Follow Up Registry Database (WEFURD), were included in the study. Prospective long-term follow-up was conducted until June 2013. The endpoints were time to treatment failure, time to seizure remission, and time to first seizure.

**Results:**

This study included 654 patients: CBZ (n=125), VPA (n=151), LTG (n=135), TPM (n=76), and OXC (n=167). The retention rates of CBZ, VPA, LTG, TPM, and OXC at the third year were 36.1%, 32.4%, 57.6%, 37.9%, and 41.8%, respectively. For time to treatment failure, LTG was significantly better than CBZ and VPA (LTG vs. CBZ, hazard ratio, [HR] 0.80 [95% confidence interval: 0.67-0.96], LTG vs. VPA, 0.53 [0.37-0.74]); TPM was worse than LTG (TPM vs. LTG, 1.77 [1.15-2.74]), and OXC was better than VPA (0.86 [0.78-0.96]). After initial target doses, the seizure remission rates of CBZ, VPA, LTG, TPM, and OXC were 63.0%, 77.0%, 83.6%, 67.9%, and 75.3%, respectively. LTG was significantly better than CBZ (1.44 [1.15-1.82]) and OXC (LTG vs. OXC, 0.76 [0.63-0.93]); OXC was less effective than LTG in preventing the first seizure (1.20 [1.02-1.40]).

**Conclusion:**

LTG was the best, OXC was better than VPA only, while VPA was the worst. The others were equivalent for comparisons between five AEDs regarding the long-term treatment outcomes of monotherapy for adult patients with focal epilepsy in a clinical practice. For selecting AEDs for these patients among the first-line drugs, LTG is an appropriate first choice; others are reservation in the first-line but VPA is not.

## Introduction

Epilepsy is a common disorder of the brain. It affects 50 million people worldwide [1]. China has about 9 million patients with epilepsy [2]. Approximately 70% epilepsy patients could attain long-term remission using currently available antiepileptic drugs (AEDs) [3]. For the most definitely diagnosed epilepsy patients, long-term treatment with AEDs is needed [4]. Monotherapy is generally recognized as the first-line treatment option for newly diagnosed patients [5, 6]. Choosing the correct or better AED may provide early relief, alleviate or reduce adverse effects, improve prognosis, and reduce financial burden in affected patients. Hence it is essential to compare long-term effectiveness of usual AEDs for monotherapy. Although there are numerous randomized controlled trials (RCTs) for evidence-based medicine [6], clinically relevant information from numerous treatment options given by fixed-dose regulatory studies is limited and the results are difficult to transfer to general practice [7–10]. The American Academy of Neurology (AAN) and International League Against Epilepsy (ILAE) Commission have recommended more meaningful long-term comparative trials that are representative of real-world clinical practices [10–12]. Epilepsy registries that were developed during the past years provide “real world” clinical practice data from prospective cohort observational studies [13–15].

Well-designed long-term clinical trials with head-to-head comparisons may provide answers to questions such as how to ensure and maintain efficacy of a new treatment over months or years and compare the long-term adverse effects of these drugs[7]. Studies have increasingly been focusing on observational studies in a real-world clinical practice setting [7–9, 16–18]. However, there are only a few studies that compare long-term treatment outcomes of AEDs as a monotherapy in epilepsy patients. For newly diagnosed epilepsy patients, the Glasgow Epilepsy Unit[19] compared the long-term pharmacological outcomes between carbamazepine (CBZ), valproate (VPA), and lamotrigine (LTG). Hu et al. (2011) [17] compared long-term effectiveness of CBZ and VPA in newly diagnosed patients with focal seizures. In another comparative study, Hu et al. (2012) [18] reported the long-term outcomes of VPA (sustained-release formulation) and topiramate (TPM) in patients with generalized or focal seizures. As a large-scale pragmatic RCT, the SANAD study compared the long-term outcomes of AEDs for monotherapy of epilepsy patients [20, 21]. Other observational pragmatic studies [8, 9, 22] compared two or three AEDs not merely for monotherapy.

There is an alarming dearth of comparative studies on long-term outcomes of AEDs in adult patients with focal epilepsy. This long-term outcome research was a prospective cohort study in adult patients with focal epilepsy registered in Wenzhou Epilepsy Follow Up Registry Database (WEFURD) to evaluate and compare long-term outcomes of monotherapy with CBZ, VPA, LTG, TPM, and oxcarbazepine (OXC) by individualized selections for monotherapy in a clinical practice setting.

## Methods

### Registry setup

Epilepsy Long-term Follow Up Registry Study (ELFURS) was a prospective, observational study for epilepsy patients in routine clinical practice, ELFURS was established at Epilepsy Unit in The First Affiliated Hospital of Wenzhou Medical University in January 2003. The study, in which signing of informed consent was waived off, was approved by the clinical research ethics committee of The First Affiliated Hospital of Wenzhou Medical University and registered in The World Health Organization (WHO) Registry Network (registration number: ChiCTR-OCH-14004616). WEFURD was established simultaneously to record, save, and process the register data. Covering a total population of nearly 10 million from Wenzhou and surrounding areas, WEFURD is the largest epilepsy database in Zhejiang Province, China. By March 2014, WEFURD has enrolled 3305 epilepsy patients.

After being definitely diagnosed with epilepsy for the first time, the patients were informed verbally about the nature of ELFURS and willingly to be recruited and the information recorded in WEFURD included patient demographics, age at onset, specific clinical manifestations of seizures, the number of seizures one year before medication, medication history about previous antiepileptic treatment, mental development situation, antecedents (history of febrile convulsions, cerebral trauma, cerebral infection, brain tumor, cerebrovascular disorders, cerebral immunologic disorders, developmental anomalies of cerebral structure, and other related neurologic conditions [23]), comorbidities, general physical examination results, and neurological examination results. Results of laboratory examinations, imaging examinations (computed tomography [CT], magnetic resonance imaging [MRI], results were classified according to ILAE recommendations[24]), electroencephalogram (EEG), and video electroencephalogram (VEEG) in following visits would be added in WEFURD.

Under the guidance for standardized recommendations [25, 26], Dr Zheng and Dr Xu chose AEDs on the basis of individual patient conditions [27]. Doses were optimized according to recommended programs and personal reactions [28]. Patients were followed up (as outpatients) every 1–3 months. Seizures were recorded in diaries by the patients or their families under the supervision of the physicians and estimated by the specialists. Important information considering prognosis and treatment effectiveness was updated in the following visits. If the patients did not visit the outpatient clinic over 6 months, we tried to contact them telephonically or via letters to encourage them to make an outpatient clinic visit. The patients were considered as lost to follow-up if the contacts were lost for more than one year. Designated two researchers to maintained WEFURD, filled information for timely update of records, and reminded the patients for monthly follow-up, if needed.

### This study protocol

This prospective cohort study was derived from ELFURS and the procedure was the same as mentioned above. The patients’ inclusion criteria, outcome evaluation, and analysis in detail were as follows.

#### Patients

The patients who were recorded from January 2004 to June 2012 in WEFURD were enrolled in this study. The last follow-up visit was in June 2013. The inclusion criteria was as follows: (1) firstly definitely diagnosed focal epilepsy patients according to classification of epileptic seizures and syndromes proposed by ILAE in 1981 and 1989 [29, 30]; (2) patients with diagnosed age ≥16 years; (3) patients who had at least two unprovoked seizures within a year before medication; (4) patients with irregular medication (before treatment in our epilepsy unit, the patients took inappropriate drugs which lack of the highest level of evidence for epilepsy/seizures [31] or without a documented attempt to titrate the dose to a target clinically effective dose range [28] even if taking an appropriate AED, and the patients should stopped medication at least for two weeks.) or without antiepileptic medication in the first visit in our unit, or those who just began to take AEDs within 4 weeks in other hospitals; (5) patients prescribed with monotherapy of one of the five AEDs: CBZ, VPA (sustained-release formulation), LTG, TPM, and OXC, all of the AEDs were immediate-release formulations except VPA. The exclusion criteria included the following: (1) patients who did not cooperate with nor had difficulty in recording seizures; (2) patients who took one AED for more than four weeks in other hospitals and were prescribed with the same AED at the first visit in our unit; (3) idiopathic focal epilepsy patients at onset in children or juvenile.

#### Outcome measures

The primary outcome of this study was time to treatment failure during three years after being prescribed with AEDs. The retention rates were calculated and the causes of treatment failure were also explored. The secondary outcomes were time to seizure remission in three years after initial target doses (ITDs) of AEDs, time to first seizure in three years after ITDs, and maximum maintenance doses (MMDs) of AEDs. The tolerability outcome was the incidence of AEs during three years after being prescribed with AEDs, which was assessed using the formula below: the number of AEs that occurred in a time period (more than one AE occurred in one patient were accumulated) divided by the number of patients taking AEDs in that period.

#### Outcome measure definition

(1) Treatment failure: discontinuation of the original AEDs, addition or replacement by another AED, undergo surgery, death caused by seizures; (2) causes of treatment failure: lack of efficacy (LE), AEs, and others (pregnancy, poor economic conditions, non-compliance (discontinuation of AEDs by themselves with a single discontinuation time of ≥ two weeks), deaths related to seizures); (3) seizure remission: “freedom from seizures for a minimum of three times the longest pre-intervention interseizure interval (determined from seizures occurring within the past 12 months) or 12 months, whichever is longer” proposed by ILAE commission [31]; (4) ITD and MMD: the daily doses which specialists planned to titrate to and maintain for at least for one visit (4 weeks) were defined as ITDs. The maximum daily doses that specialists prescribed as clinically effective and appropriate and maintain for more than 4 weeks, and with which the patients could tolerate were defined as MMDs. Two weeks after being prescribed with ITDs and MMDs, the patients were regarded as achieving ITDs and MMDs according to the pharmacokinetics of the five AEDs [32] studied in this report.

#### Statistical analysis

SPSS17.0 for Windows was used to perform all statistical analysis. Continuous variables of skewness distribution were summarized using the median, interquartile range (IQR), and range. Categorical variables were summarized using counts and percentages. Retention rate and seizure remission rate were estimated using Kaplan-Meier survival analysis.

The baseline demographic and clinical characteristics of different AEDs groups may not be balanced because specialists chose AEDs individually [27]. Selection bias could not be avoided in comparing effectiveness of AEDs in clinical practice. Therefore potential confounding factors should be excluded in using Cox regression model during multivariate analysis, allowing for a more scientific comparison of different AEDs [18, 33]. Cox proportional hazards models (95% confidence interval [CI]) were employed to analyze the hazard ratios (HRs). The confounding factors analyzed are listed in Table 1. In analyzing time to first seizure after ITDs, the factor of PDD/DDD ratio was excluded from adjusting for potential confounding factors. Kruskal-Wallis H test was used to compare the overall distribution of PDD/DDD ratios. Chi-square test was used to compare the incidences of AEs, and the other outcome measures were compared using Cox regression model.

**Table 1:** Baseline clinical characteristics and demographic information.

	carbamazepine	valproate	lamotrigine	topiramate	oxcarbazepine	Total
**N**	125	151	135	76	167	654
**Male** ^a^ **(%)**	67(53.6)	86(57.0)	51(37.8)	39(51.3)	76(45.5)	319(48.8)
**Age** ^a^ **, median(range) (years)**	29(16–72)	27(16–88)	31(16–60)	28(16–64)	33(16–71)	29(16–88)
**Age at seizure onset** ^a^ **, median(IQR) (years)**	23(15)	21(14)	23(20)	26(17)	25(22)	23(17)
**Seizure duration** ^a^ **, median(IQR)(years)**	4(9.1)	3(7.3)	3(9.0)	2(5.1)	3(9.5)	3(9.3)
**History of irregular medication** ^a^ **, (%)**	65(52.0)	59(39.1)	50(37.0)	32(42.1)	77(46.1)	283(43.3)
**Seizures within the past one year** ^a^ **(%)**						
**≤20**	76(60.8)	98(64.9)	91(67.4)	60(78.9)	87(52.1)	412(63.0)
**>20**	49(39.2)	53(35.1)	44(32.6)	16(21.1)	80(47.9)	242(37.0)
**The longest pre-intervention interseizure interval of one year before medication** ^a^ **(months) (%)**						
**≤4**	100	108	92	51	142	493
**>4**	25	43	43	25	25	161
**Seizure type** ^a^ **(%)**						
**Simple or complex partial seizure only**	33(26.4)	20(13.2)	30(22.2)	6(7.9)	34(20.4)	123(18.8)
**Secondary generalized tonic-clonic seizure**	92(73.6)	131(86.8)	105(77.8)	70(92.1)	133(79.6)	531(81.2)
**Etiological classification of epilepsy** ^a^ **(%)**						
**Symptomatic**	91(72.8)	75(49.7)	74(54.8)	39(51.3)	83(49.7)	362(55.4)
**Cryptogenic**	32(27.2)	76(50.3)	61(45.2)	37(48.7)	84(50.3)	292(44.6)
**Imagining (CT or MRI result) (%)**					
**Related abnormal**	56(44.8)	35(28.0)	40(29.6)	19(25.0)	43(25.7)	195(29.8)
**Normal**	55(44.0)	92(60.9)	80(59.3)	50(65.8)	114(68.3)	391(59.8)
**Loss or not done**	14(11.2)	24(15.9)	15(11.1)	7(9.2)	8(4.8)	68(10.4)
**Antecedents** ^a^ **(%)**	60(48.0)	53(35.1)	50(37.0)	25(32.9)	47(28.1)	235(35.9)
**ITD, range (mg/day)**	300–800	500–1500	50–200	75–250	450–1800	/
**MMD** ^b^ **(range) (mg/day)**	600 (300–1200)	1000 (500–2000)	100 (50–250)	200 (75–400)	900 (450–2400)	/
**PDD/DDD ratio** ^a^ ^**,**^ ^c^ **, median(IQR)**	1.0(0.7)	1.0(0)	0.7(0.7)	1.2(0.6)	1.0(0.3)	1(0.4)

IQR: interquartile range; CT: computed tomography; MRI: magnetic resonance imaging; ITD: initial target dose; MMD: maximum maintenance dose.

^a^ Confounders which were included in the Cox proportional hazard models.

^b^ MMD: the data of patients who had treatment failure before being titrated to initial target dose was excluded.

^c^ PDD: prescribed daily dose. The MMDs were adopted as PDDs, and in patients who withdrew before being titrated to ITDs, the maximum doses that had been taken were adopted. DDD: defined daily dose, 1 DDD = 600 mg [CBZ], 1000 mg [VPA], 135 mg [LTG], 170 mg [TPM], 950 mg [OXC]. The principle of establishing the DDDs was to have a similar distribution of PDD/DDD ratios between each AED in pairwise comparison to make it to possible to compare the use conditions of AEDs

**Fig 1:**
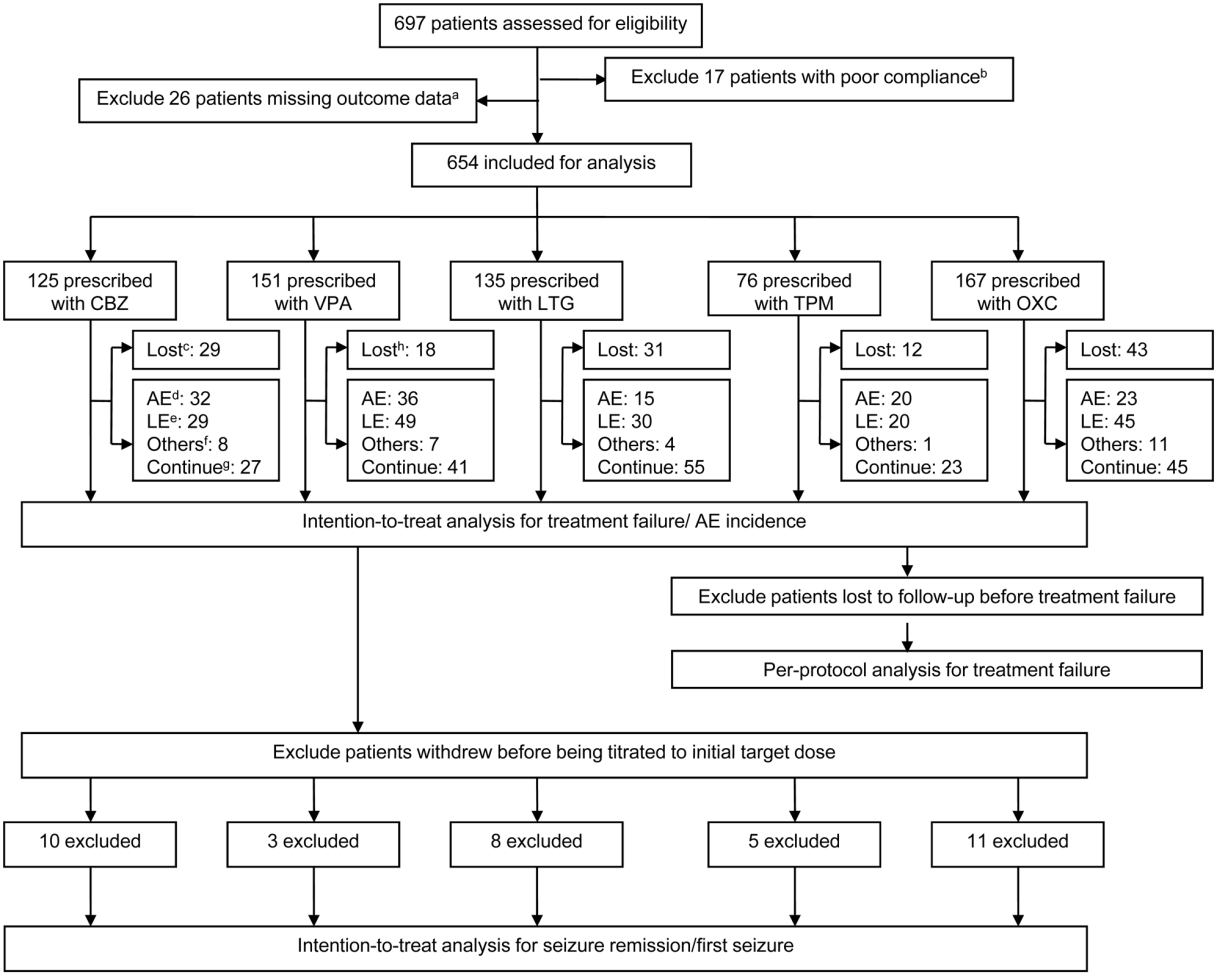
Study flow diagram. ^a^patients missing outcome data: patients who did not complete effectiveness/safety assessment at least for one time after being prescribed with AEDs; ^b^patients with poor compliance: patients who discontinued AEDs by themselves with a single discontinuing time less than two weeks but the accumulated one >20% of the total observational period. Patients who did not take AEDs according to the prescribed doses and the doses they took were also lower than the initial target doses (ITDs); ^c^Lost: lost to follow-up before treatment failure; ^d^AEs: treatment failure because of adverse events; ^e^LE: treatment failure because of lack of efficacy; ^f^Others: other reasons leading to treatment failure: pregnancy, poor economic conditions, non-compliance and deaths related to seizures; ^g^Continue: continue treatment on AEDs; ^h^Lost: including one person who died without relation to AED medication; CBZ: carbamazepine; VPA: valproate; LTG: lamotrigine; TPM: topiramate; OXC: oxcarbazepine.

Intention-to-treat (ITT) analysis was used to analyze the primary and tolerability outcome, and primary outcome was also analyzed using per-protocol (PP) analysis whose population was defined as the remaining one after excluding patients who were lost to follow-up before treatment failure in ITT analysis(The analysis population is shown in Fig 1). The censored patients in Kaplan-Meier analysis and Cox regression were defined as follows: patients who were lost to follow-up, or died but whose death were not related to AED medication before their outcomes (treatment failure, seizure remission, and first seizure) were observed and the outcomes were still not observed at the last visit of this study (for primary and secondary outcomes) or when treatment failure occurred (only for secondary outcomes). The statistical significance level was set at P = 0.05 using two-sided tests.

## Results

### Baseline characteristics

Table 1 shows the baseline characteristics and demographic information of the five AEDs groups. 654 patients (48.8% men) were included in this study: 125 for CBZ, 151 for VPA, 135 for LTG, 76 for TPM, and 167 for OXC. Median duration of follow-up before treatment failure was 11.4 (IQR, 3.6–32.9) months, 12.7 (4.0–29.7) months, 18.1 (5.4–32.2) months, 20.6 (4.8–36.0) months and 14.1 (6.1–29.4) months respectively. Cox proportional hazards models showed that confounding bias in comparing retention rates were as follows: seizure duration (HR 1.03 [95%CI: 1.01–1.04]), more than 20 seizures one year before medication (1.87 [1.49–2.34]) were the risk factors, and the maximal dose (PDD/DDD ratio) (0.62 [0.43–0.89]) during treatment was the protective factor.

### Outcome measures

#### Time to treatment failure

**Fig 2:**
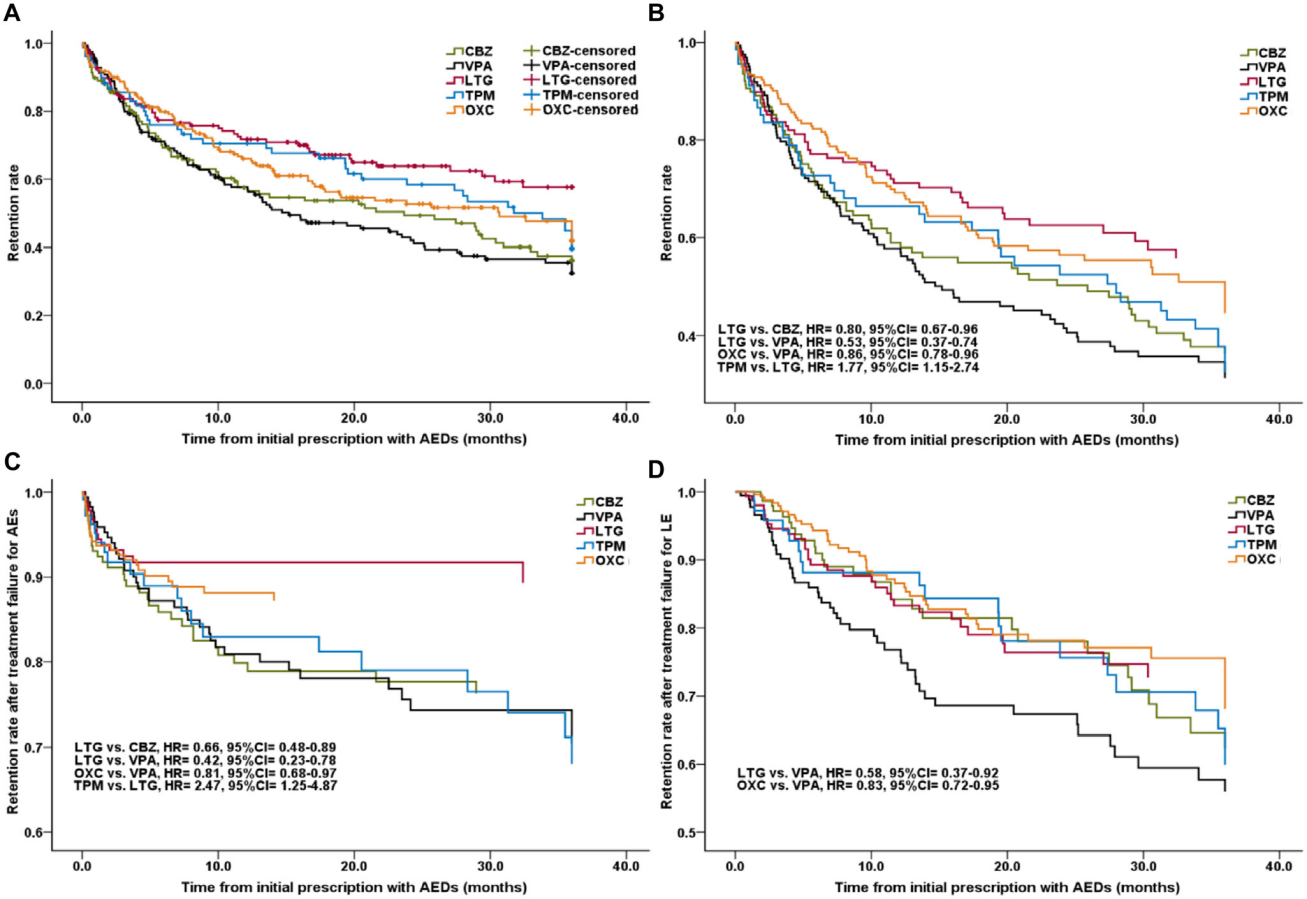
Retention rates of AEDs. Overall retention rate by Kaplan-Meier analysis (A) and Cox regression (B); Retention rate after treatment failure for AEs (C) and LE (D) by Cox regression; CBZ: carbamazepine; VPA: valproate; LTG: lamotrigine; TPM: topiramate; OXC: oxcarbazepine; AED: antiepileptic drug; AE: adverse event; LE: lack of efficacy; HR: hazard ratio; CI: confidence interval.

In the ITT analysis, the retention rates of CBZ, VPA, LTG, TPM, and OXC at the third year were 36.1%, 32.4%, 57.6%, 37.9%, and 41.8%, respectively (Fig 2A). For time to treatment failure, after adjustment for potential confounders by Cox proportional hazards models, LTG was significantly better than CBZ, VPA, and TPM (LTG vs. CBZ, 0.80 [0.67–0.96]; LTG vs. VPA, 0.53 [0.37–0.74]; TPM vs. LTG, 1.77 [1.15–2.74]). OXC was also better than VPA (0.86 [0.78–0.96]). There were no differences in pairwise comparison of retention rates between other AEDs (Fig 2B). The PP analysis showed similar results (LTG vs. CBZ, 0.78 [0.65–0.94]; LTG vs. VPA, 0.60 [0.42–0.85]; TPM vs. LTG, 1.67 [1.08–2.59]) except the comparison between OXC and VPA (OXC vs. VPA, 0.93 [0.84–1.02]) (S1 Fig).

#### Causes of treatment failure

Table 2 shows the proportion of different reasons leading to treatment failure. For ITT analysis, CBZ, VPA, and TPM showed lower retention rates comparing with LTG because of the lesser association of LTG with treatment failure for AEs (LTG vs. CBZ, 0.66 [0.48–0.89]; LTG vs. VPA, 0.42 [0.23–0.78]; TPM vs. LTG, 2.47 [1.25–4.87]). This treatment failure also occurred less in OXC than VPA (0.81 [0.68–0.97]) leading to lower retention rate of VPA. No statistical differences were found between other pairwise comparisons (Fig 2C). Cox regression analysis also showed that LTG and OXC had higher retention rates than VPA for lower proportion of treatment failure due to LE (LTG vs. VPA, 0.59 [0.37–0.94]; OXC vs. VPA, 0.82 [0.72–0.95]), and there were no significant differences between other pair-wise comparisons of AEDs (Fig 2D).

**Table 2:** Reasons for treatment failure.

	carbamazepine	valproate	lamotrigine	topiramate	oxcarbazepine
N	125	151	135	76	167
Adverse events	32(25.6)	36(23.8)	15(11.1)	20(26.3)	23(13.8)
Lack of efficacy	29(23.2)	49(32.5)	30(22.2)	20(26.3)	45(26.9)
Others^a^	8(6.4)	7(4.6)	4(3.0)	1(1.3)	11(6.6)

^a^Others: other reasons including pregnancy, poor economic conditions, non-compliance (discontinuation of AEDs by themselves with a single discontinuation time of ≥ two weeks), deaths related to seizures

#### Time to seizure remission

**Fig 3:**
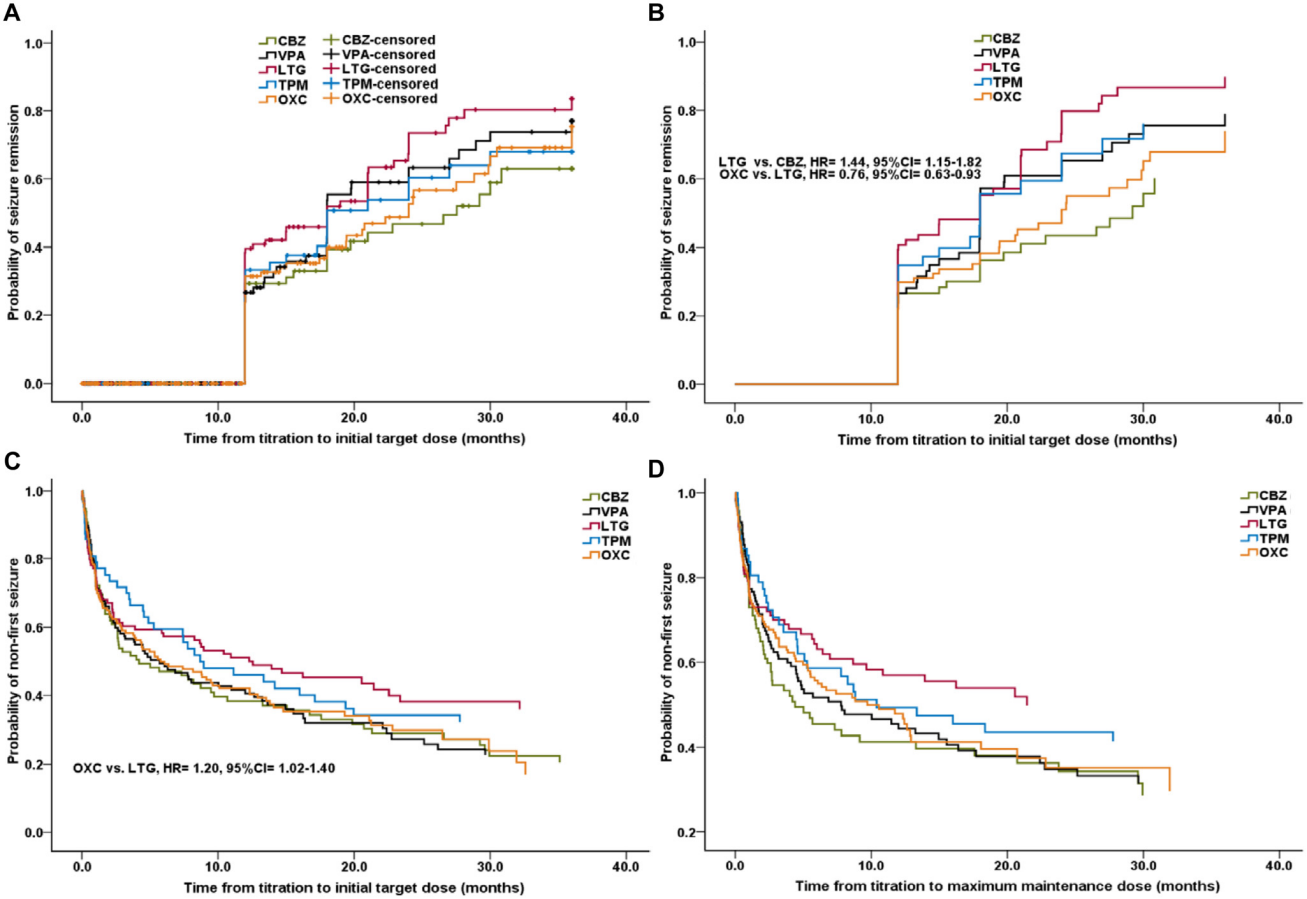
The secondary outcomes of AEDs. Time to seizure remission after titrating to initial target dose by Kaplan-Meier analysis (A) and Cox regression (B); Time to first seizure after titrating to initial target dose (A) and maximum maintenance dose (B) by Cox regression; CBZ: carbamazepine; VPA: valproate; LTG: lamotrigine; TPM: topiramate; OXC: oxcarbazepine; AED: antiepileptic drug; HR: hazard ratio; CI: confidence interval.

After titrating to ITDs of AEDs, there was one patient who could not be observed for 12 months till last follow-up visit, so he was observed for more time till 12 months to meet the requirements to assess seizure remission. The seizure remission rates were 63.0%, 77.0%, 83.6%, 67.9%, and 75.3% for CBZ, VPA, LTG, TPM, and OXC after achieving ITDs in three years, respectively (Fig 3A). By Cox regression analysis, LTG was more likely to have a seizure remission than both CBZ and OXC (LTG vs. CBZ, 1.44 [1.15–1.82]; OXC vs. LTG, 0.76 [0.63–0.93]). Other pair-wise comparisons showed no significance (Fig 3B).

#### Time to first seizure

By Cox regression analysis, after titrating to ITDs, LTG was more effective in preventing first seizures than OXC (OXC vs. LTG, 1.20 [1.02–1.40]). Pair-wise comparisons showed no other differences (Fig 3C). After MMDs were attained, no significant differences were found between each AED (Fig 3D).

#### Tolerability

**Fig 4:**
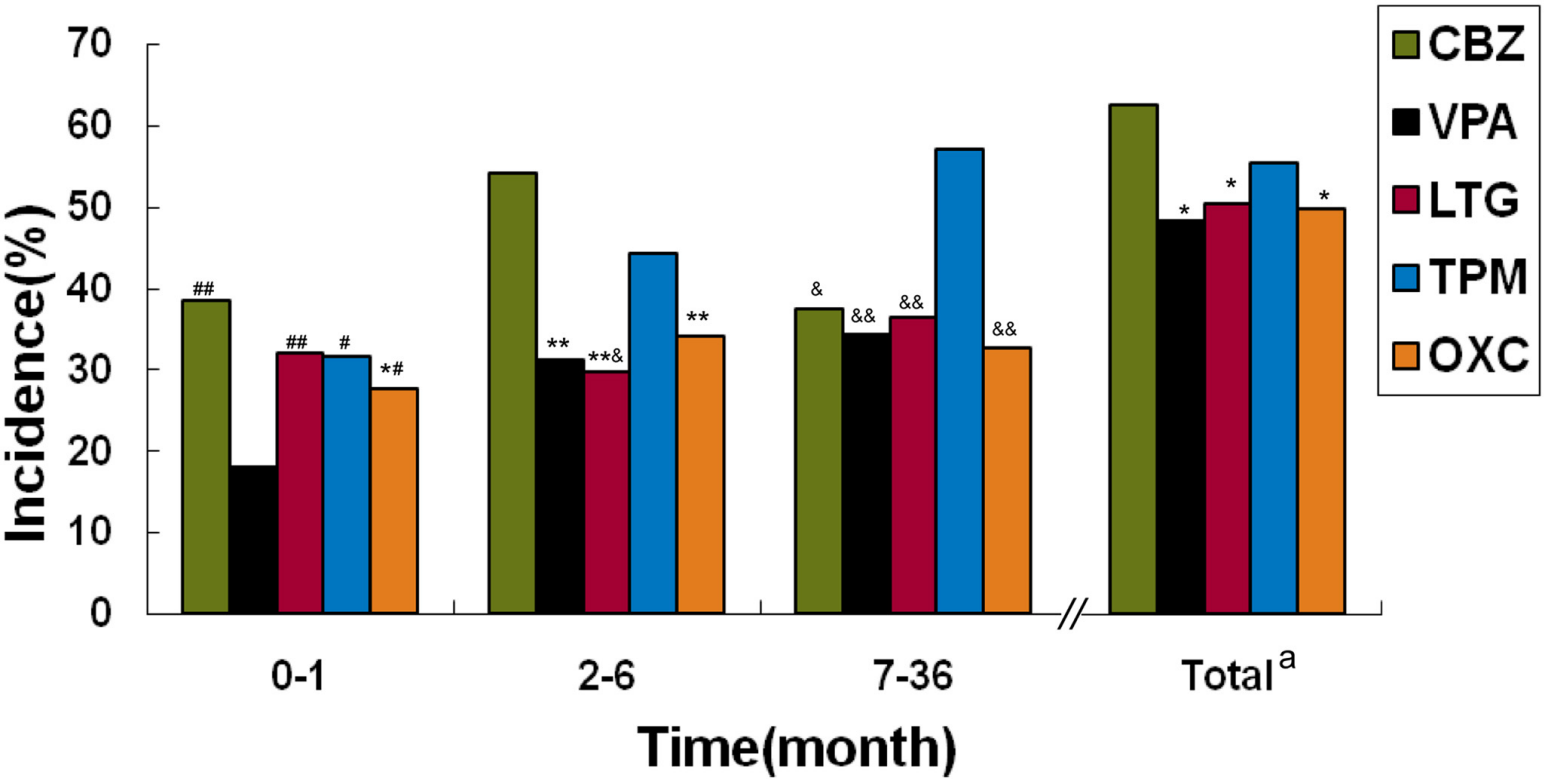
Incidence of adverse events during follow-up. ^a^ Total: total percentage of patients with at least one adverse event; compared with CBZ, *: P<0.05, **: P<0.01; compared with VPA, #: P<0.05, ##: P<0.01; compared with TPM, &: P<0.05, &&: P<0.01; CBZ: carbamazepine; VPA: valproate; LTG: lamotrigine; TPM: topiramate; OXC: oxcarbazepine.

Overall, in terms of the percentage of patients with at least one AE in the 3-year observation period, CBZ was higher than VPA, LTG, and OXC (all P<0.05), and other paired comparisons showed no significant differences (Fig 4). Table 3 shows the AEs of each AED during three years. The neuropsychiatric symptoms whose common individual ones included dizziness/vertigo, memory deterioration, and insomnia were the most common in the five AEDs. For the individual symptoms, rash was a common early phase symptom with CBZ, LTG, and OXC; gastrointestinal reactions were very prevalent in each individual drug; liver dysfunction was a common AE of CBZ and VPA. Other common AEs were cytopenia (common with CBZ and VPA), weight change (common with VPA and TPM), kidney calculus (common with TPM), numbness (common with TPM), and tremor (common with VPA). In the first month (early phase), the AE incidence of VPA was the lowest, significantly lower than other four drugs (VPA vs. CBZ/LTG, P<0.01; VPA vs. TPM/OXC, P<0.05). The AE incidence of OXC was lower than that of CBZ (P<0.05), which was the highest in the early phase, and there were no significant difference between other pairwise comparisons. In 2–6 months (medium-term), the AE incidence of CBZ was the highest, significantly higher than that of VPA, LTG, and OXC (all P<0.01), and the one of TPM was also higher than that of LTG (P<0.05). No other paired comparisons of medium-term AE incidence showed a significant difference. In the 7–36 month interval (long-term), the AE incidence of TPM was the highest, all significantly higher than that of other drugs (TPM vs. VPA/LTG/OXC, P<0.01; TPM vs. CBZ, P<0.05) (Fig 4).

**Table 3:** Adverse events n (%).

Body system	carbamazepine	valproate	lamotrigine	topiramate	oxcarbazepine
**n**	**125**	**151**	**135**	**76**	**167**
**Total events** ^a^	**78(62.4)**	**72(48.0)**	**68(50.4)**	**42(55.3)**	**83(49.7)**
**Neuropsychiatric system**	**64(51.2)**	**36(23.8)**	**55(40.7)**	**51(67.1)**	**76(45.5)**
Dizziness/vertigo	14(11.2)	3(2.0)	9(6.7)	5(6.6)	36(21.6)
Hypomnesia	11(8.8)	5(3.3)	13(9.6)	12(15.8)	19(11.4)
Headache	10(8.0)	1(0.7)	11(8.1)	5(6.6)	5(3.0)
Drowsiness	8(6.4)	7(4.6)	4(2.9)	1(1.3)	4(2.4)
Insomnia	8(6.4)	4(2.6)	12(8.8)	9(11.8)	7(4.2)
Tremor	1(0.8)	10(6.6)	1(0.7)	0	0
Numbness	6(4.8)	2(1.3)	2(1.5)	14(18.4)	0
Slow response	2(1.6)	1(0.7)	0	2(2.6)	0
Anxiety/ depressive disorder	0	1(0.7)	3(2.2)	0	2(1.2)
Other neuropsychiatric symptoms^b^	4(3.2)	2(1.3)	0	3(3.9)	3(1.8)
**Skin system**	**15(12.0)**	**5(3.3)**	**22(16.3)**	**6(2.6)**	**14(5.4)**
Skin rash	11(8.8)	0	19(14.1)	1(1.3)	13(7.8)
Hair loss	0	5(3.3)	2(1.5)	3(3.9)	1(0.6)
Other skin symptoms^c^	4(3.2)	0	1(0.7)	2(2.6)	0
**Digestive system**	**24(19.2)**	**32(21.2)**	**14(10.4)**	**9(11.8)**	**11(6.6)**
Liver dysfunction	13(10.4)	13(8.6)	1(0.7)	0	2(1.2)
Gastrointestinal reactions^d^	11(8.8)	19(12.6)	13(9.6)	9(11.8)	9(5.4)
**Hemic system**	**8(6.4)**	**3(2.0)**	**3(2.2)**	**0**	**1(0.6)**
Cytopenia^e^	8(6.4)	3(2.0)	1(0.7)	0	1(0.6)
Epistaxis / Gum bleeding	0	0	2(1.5)	0	0
**Fatigue/tiredness**	**8(6.4)**	**3(2.0)**	**3(2.2)**	**2(2.6)**	**13(7.8)**
**Blurred vision**	**2(1.6)**	**3(2.0)**	**1(0.7)**	**2(2.6)**	**6(3.6)**
**Weight gain**	**1(0.8)**	**14(9.3)**	**4(2.9)**	**0**	**1(0.6)**
**Weight loss**	**1(0.8)**	**0**	**2(1.5)**	**8(10.5)**	**0**
**Menstrual disorder**	**1(0.8)**	**4(2.6)**	**2(1.5)**	**0**	**0**
**Physical pain**	**1(0.8)**	**1(0.7)**	**1(0.7)**	**2(2.6)**	**5(3.0)**
**Kidney stone**	**0**	**0**	**0**	**6(7.9)**	**0**
**Other events** ^f^	**3(2.4)**	**4(2.6)**	**8(5.9)**	**1(1.3)**	**12(7.2)**

^a^ Total events: total patients with at least one adverse event;

^b^ Other neuropsychiatric symptoms included language disorders, ataxia, hearing decrease, impairments of calculation and irritability;

^c^ Other skin symptoms included hypohidrosis and decrustation;

^d^ Gastrointestinal reactions included vomiting, nausea, and abdominal discomfort;

^e^ Cytopenia included leucopoenia, oligocythemia, and thrombocytopenia;

^f^ Other events included fever, lower limbs edema, tinnitus, renal dysfunction, palpitation, ostealgia/arthralgia, hyperdacryosis, upper respiratory infection, oral ulcer, chest stufly and bitter taste.

## Discussion

Studies from registries and RCTs had important and complementary effects in evaluating patient outcomes [34]. Epilepsy and pregnancy registries performed internationally were excellent works so far [35]; discovering a number of teratogenic effects of AEDs on human beings and changing patterns of AED use in pregnant women, thereby benefitting them [36]. Some findings were also published by UK AED Register [13, 15]. Our prospective cohort study fills the vacancy with evidence of comparisons between five common AEDs regarding long-term outcomes in real-world practice, making sense for selection of AEDs as monotherapy for adult focal epilepsy patients.

As recommended, AED study design should be performed in real-world settings, providing comprehensive measures of effectiveness, efficacy, and tolerability [10]. This prospective cohort study basically conformed to the above requirements with adult focal epilepsy patients as the study objects receiving monotherapy in routine clinical practice could stand for population from Wenzhou and surrounding areas and significant clinical comprehensive measures with long-term epilepsy follow-up outcomes were employed.

For time to treatment failure, we found that LTG was less likely than CBZ, VPA, and TPM to have treatment failure. OXC was also less likely than VPA to fail in ITT analysis. SANAD study [20] found that LTG was less likely to be withdrawn than CBZ and TPM, OXC showed a similar performance in this aspect with LTG and TPM, and CBZ was similar to TPM too. An observational study performed by Hu et al. (2011) [17] did not find significant difference of retention rates between CBZ and VPA as the initial monotherapy in patients with newly diagnosed focal seizures. Those findings [17, 20] were consistent with our findings. Another study reported by Hu et al. (2012) [18] showed that VPA was better than TPM for time to treatment failure in treating patients with focal and generalized tonic-clonic seizures (GTCS). It was potentially related to idiopathic epilepsy patients with only GTCS, who were more suitable to VPA treatment [5, 21]. Consequently, the retention of VPA was increased in the study by Hu et al. (2012) [18].

AEs and LE were the main reasons for treatment failure of AEDs; their performances varied according to different drugs. LTG was better than CBZ, VPA, and TPM for rarely had treatment failure due to AEs in our study. OXC was also less likely to produce this treatment failure than VPA. Hu et al. reported VPA had similar performance with TPM [18] or CBZ [17] for treatment failure because of AEs. LTG was less likely than CBZ and TPM to fail because of AEs in SANAD study [21]. The mentioned results above [7, 9, 17, 18, 20] were in accordance with our results. This study also showed that VPA was more likely than LTG and OXC to fail for LE, while no significant differences were observed between other paired comparisons. This finding seemed to contrast with observational studies, in which VPA was less likely than CBZ [17] and TPM [18] to have this treatment failure. However, the value was nearly identical, with P = 0.047 compared with CBZ [17]. On comparing VPA to TPM, idiopathic epilepsy patients with GTCS only were included [18]. The superior efficacy of VPA in treating this type of patients than focal epilepsy patients [19] led to the difference between Hu et al. (2012) study [18] and our study. SANAD study [20] showed that TPM was more likely than CBZ to fail because of LE. This result from an RCT [20] was not consistent with our study results.

As Mohanraj and Brodie [37] had pointed out, one potential deficiency in newly diagnosed epilepsy was excluding the possibility that any difference, which was caused by differences in titration schedules or maintenance dosing, between the standard and new drugs in terms of time to first seizure. Our study took into consideration their points of view [37] to observe the first seizure after being titrated with ITDs and MMDs. After being titrated to ITDs, OXC was more likely to have first seizure than LTG. It could be because the conservative ITDs caused by limited tolerability of a part of patients on OXC took leading OXC to be less effective to prevent first seizure than LTG in our study. After being titrated to MMDs, our report found that AEDs showed no significant difference to each other, suggesting that each AED could be obtained similar performance in preventing first seizure as long as MMDs were attained.

In the initial phase of medication, AEDs should be titrated to ITDs, causing unsteady effective blood concentration during the titration course. Therefore, it was improper to evaluate time from the beginning of medication to seizure remission. Just as ILAE pointed out, the outcome of intervention should be evaluated at adequate strength/dosage for sufficient duration [31]. Our study evaluated time to seizure remission after ITDs and the new definition of seizure remission of unequivocal effects of intervention in drug-resistant epilepsy [31] was adopted. According to this definition [31], we found that LTG was more effective than CBZ and OXC to have seizure remission. Previous study performed showed that VPA had similar time to 12-month remission with TPM [18], and SANAD study [20] did not show any difference between CBZ, LTG, TPM, and OXC in this regard. What called for special attention was that the percentage of 12-month remission of LTG showed inferiority to superiority than CBZ as time progress [20], demonstrating the possibility of a better performance on seizure remission than CBZ. Glasgow epilepsy unit [19] also found that LTG had higher 12-month seizure freedom rate than CBZ for focal epilepsy patients. This result [19] was consistent with our findings. This study also found that OXC, which was derived from CBZ and maintain equal efficacy [38], was less likely to have seizure remission than LTG, showing better ability to have remission of LTG than CBZ on the other side.

Treatment-related adverse effects strongly correlated with quality of life of patients [39]. The success or failure of a drug was mainly determined by its side effect profile [9] and the most important outcome measure was withdrawal of a drug for intolerable or fatal side effects [37]. The incidence of AEs in CBZ was the highest in three years, but the percentage of patients who discontinued treatments because of AEs was equal to VPA and TPM. This obviously resulted not from the highest treatment failure rate of patients with AEs who took CBZ, while more patients with AEs taking TPM or VPA were more likely to have their treatment discontinued (S1 Table). On the whole, LTG showed the best safety excluding rash, OXC had better tolerability than CBZ. In long-term phase, TPM had the highest AE incidence, AEs including kidney calculus and cognitive impairments should be paid attention to while using TPM [9, 40].

Our data, which was extracted from ELFURS with relative loose inclusion and exclusion criteria, was well representative of general patients in clinical practice and its analysis results showed strong external applicability/validity. Ours was a meaningful work, as UK AED Register [13] pointed out that the register cannot replace an RCT, but the body of evidence of the burden on AEDs can be added by a register, and the information from the registers was valuable and perhaps even more applicable for the AEDs used reflecting the current clinical practice. We will continue to give trail reports about research results of our unit in the subsequent studies.

### Limitations

The lack of randomization of an observational cohort study may increase the risk of imbalance across treatment groups in important variables. To compensate for this disadvantage, Cox regression models were used to adjust for as many potential confounders as possible. It had been reported that dosing titration speed would have an effect on discontinuation of treatment[41]. As all the patients were treated by the only two specialists and the dosage titration speed was strictly referred to the recommendation, the dosing schedules could be relatively homogenous.

It might raise doubt that prior exposure would influence which medication was selected and the seizure outcome could be quite different if a specific medication was used as a first-line or second-line agent for around 43% of the patients who had a history of irregular treatment. But most of them took Chinese traditional medicines, even some medicines were adulterated with inconstant content of standard AEDs (phenytoin or phenobarbital) illegally [42]. Furthermore, though appropriate AEDs had been chosen by a substantial part of these patients, the quite low dose that had been taken could not evaluate the efficacy. It would not influence the decision making in the subsequent treatment that the first monotherapy could be considered as for these patients.

In prospective registry studies, patients were easily lost to follow-up because of the long observation period [17, 18, 43]. We took active measures to try to connect with patients to solve this problem, whereas a quite proportion (accounting for 20.3% of the total patients) lost inevitably. The PP analysis was performed to find the influence of the lost patients on the results. Though it should be treated with caution that the PP analysis didn’t show significant difference between OXC and VPA for primary outcomes, the other pairwised comparisons in PP analysis had similar results with that in the ITT analysis.

Though relative smaller sample size of TPM comparing to the other groups, it was verified that 85% or 90% power still could be achieved at 0.05 significance level to detect a hazard ratio of 1.52 in pair-wise comparisons according to the formula proposed by Hsieh and Lavori [44].

### Conclusion

This prospective cohort study shows the comprehensive evaluation for monotherapy outcomes of five AEDs in adult focal epilepsy patients during three years’ follow-up. The findings suggest that LTG is the best, OXC better than VPA only, while VPA is the worst; the others were equivalent for comparisons between five AEDs regarding the long-term treatment outcomes of monotherapy for those patients in clinical practice. It has been proved that LTG is an appropriate first choice, others are reservation in the first-line drugs but VPA not because of lacking long-term effectiveness. The evidence from this study provides some inferences for selecting drugs in monotherapy for adult patients with focal epilepsy in daily clinical practice.

## Supporting Information

S1 Fig Retention rates of AEDs by Cox regression in per-protocol analysis. CBZ: carbamazepine; VPA: valproate; LTG: lamotrigine; TPM: topiramate; OXC: oxcarbazepine; AED: antiepileptic drug; HR: hazard ratio; CI: confidence interval.
(TIF)

S1 Table Treatment failure rate of patients with AEs. AE: adverse event; compared with lamotrigine, *: P<0.05, **: P<0.01; compared with oxcarbazepine, #: P<0.05, ##: P<0.01;
(DOCX)

## References

[1] World Health Organization. Epilepsy; 2014 [cited 2014 15 Feb]. Available: http://www.who.int/mediacentre/factsheets/fs999/en/.

[2] Chinese Medical Association. Book for epilepsy in China clinical practice guidelines series-author's translation. 1st ed Beijing: People's Medical Publishing house 2007.

[3] Brodie MJ, Barry SJE, Bamagous GA, Norrie JD, Kwan P. Patterns of treatment response in newly diagnosed epilepsy. Neurology. 2012;78(20):1548–1554. 10.1212/WNL.0b013e3182563b19 22573629 PMC3348850

[4] Perucca E. Established antiepileptic drugs. Bailliere's clinical neurology. 1996;5(4):693–722. 9068876

[5] National Institute for Clinical Excellence. The epilepsies: the diagnosis and management of the epilepsies in adults and children in primary and secondary care2012 [cited 2014 Mar 23]; 2014(Mar 23). Available: http://www.nice.org.uk/guidance/cg137/resources/guidance-the-epilepsies-the-diagnosis-and-management-of-the-epilepsies-in-adults-and-children-in-primary-and-secondary-care-pdf.

[6] Glauser T, Ben-Menachem E, Bourgeois B, Cnaan A, Guerreiro C, Kalviainen R, et al Updated ILAE evidence review of antiepileptic drug efficacy and effectiveness as initial monotherapy for epileptic seizures and syndromes. Epilepsia. 2013;54(3):551–563. 10.1111/epi.12074 23350722

[7] Zaccara G, Messori A, Cincotta M, Burchini G. Comparison of the efficacy and tolerability of new antiepileptic drugs: what can we learn from long-term studies? Acta Neurol Scand. 2006;114(3):157–168. 10.1111/j.1600-0404.2006.00705.x 16911343

[8] Chung S, Wang N, Hank N. Comparative retention rates and long-term tolerability of new antiepileptic drugs. Seizure-Eur J Epilep. 2007;16(4):296–304. 10.1016/j.seizure.2007.01.004 17267243

[9] Bootsma HP, Ricker L, Hekster YA, Hulsman J, Lambrechts D, Majoie M, et al The impact of side effects on long-term retention in three new antiepileptic drugs. Seizure-Eur J Epilep. 2009;18(5):327–331. 10.1016/j.seizure.2008.11.006 19110447

[10] Ben-Menachem E, Sander JW, Privitera M, Gilliam F. Measuring outcomes of treatment with antiepileptic drugs in clinical trials. Epilepsy Behav. 2010;18(1–2):24–30. 10.1016/j.yebeh.2010.1004.1001 Epub 2010 May 1013. 20462803

[11] French JA, Kanner AM, Bautista J, Abou-Khalil B, Browne T, Harden CL, et al Efficacy and tolerability of the new antiepileptic drugs I: treatment of new onset epilepsy: report of the Therapeutics and Technology Assessment Subcommittee and Quality Standards Subcommittee of the American Academy of Neurology and the American Epilepsy Society. Neurology. 2004;62(8):1252–1260. 15111659 10.1212/01.wnl.0000123693.82339.fc

[12] French JA, Kanner AM, Bautista J, Abou-Khalil B, Browne T, Harden CL, et al Efficacy and tolerability of the new antiepileptic drugs II: treatment of refractory epilepsy: report of the Therapeutics and Technology Assessment Subcommittee and Quality Standards Subcommittee of the American Academy of Neurology and the American Epilepsy Society. Neurology. 2004;62(8):1261–1273. 15111660 10.1212/01.wnl.0000123695.22623.32

[13] Andrew T, Milinis K, Baker G, Wieshmann U. Self reported adverse effects of mono and polytherapy for epilepsy. Seizure-Eur J Epilep. 2012;21(8):610–613. 10.1016/j.seizure.2012.06.013 22795388

[14] Bergin P, Sadleir L, Legros B, Mogal Z, Tripathi M, Dang N, et al An international pilot study of an internet-based platform to facilitate clinical research in epilepsy: The EpiNet project. Epilepsia. 2012;53(10):1829–1835. 10.1111/j.1528-1167.2012.03636.x 22958055

[15] Wieshmann UC, Baker GA. Self-reported feelings of anger and aggression towards others in patients on levetiracetam: data from the UK antiepileptic drug register. BMJ Open. 2013;3(3).(pii):e002564 002510.001136/bmjopen-002013-002564. 10.1136/bmjopen-2013-002564 23516271 PMC3612797

[16] Sander JW. New antiepileptic drugs in practice—how do they perform in the real world? Acta Neurol Scand. 2005;112:26–29. 10.1111/j.1600-0404.2005.00505.x 16238705

[17] Hu Y, Huang Y, Quan F, Hu Y, Lu Y, Wang X-F. Comparison of the retention rates between carbamazepine and valproate as an initial monotherapy in Chinese patients with partial seizures: A ten-year follow-up, observational study. Seizure-Eur J Epilep. 2011;20(3):208–213. 10.1016/j.seizure.2010.11.020 21159526

[18] Hu Y, Huang X, Shen D, Ding M, Sun H, Peng B, et al Outcomes of sustained-release formulation of valproate and topiramate monotherapy in patients with epilepsy: a multi-centre, cohort study. PLoS One. 2012;7(12):e47982 47910.41371/journal.pone.0047982. Epub 0042012 Dec 0047911. 10.1371/journal.pone.0047982 23239963 PMC3519782

[19] Mohanraj R, Brodie MJ. Pharmacological outcomes in newly diagnosed epilepsy. Epilepsy Behav. 2005;6(3):382–387. 10.1016/j.yebeh.2005.01.008 15820347

[20] Marson AG, Al-Kharusi AM, Alwaidh M, Appleton R, Baker GA, Chadwick DW, et al The SANAD study of effectiveness of carbamazepine, gabapentin, lamotrigine, oxcarbazepine, or topiramate for treatment of partial epilepsy: an unblinded randomised controlled trial. Lancet. 2007;369(9566):1000–1015. 10.1016/s0140-6736(07)60460-7 17382827 PMC2080688

[21] Marson AG, Al-Kharusi AM, Alwaidh M, Appleton R, Baker GA, Chadwick DW, et al The SANAD study of effectiveness of valproate, lamotrigine, or topiramate for generalised and unclassifiable epilepsy: an unblinded randomised controlled trial. Lancet. 2007;369(9566):1016–1026. 10.1016/s0140-6736(07)60461-9 17382828 PMC2039891

[22] Simister RJ, Sander JW, Koepp MJ. Long-term retention rates of new antiepileptic drugs in adults with chronic epilepsy and learning disability. Epilepsy Behav. 2007;10(2):336–339. 10.1016/j.yebeh.2006.12.005 17267290

[23] Shorvon SD. The etiologic classification of epilepsy. Epilepsia. 2011;52(6):1052–1057. 10.1111/j.1528-1167.2011.03041.x 21449936

[24] Berkovic S, Duncan J, Barkovich A, Cascino G, Chiron C, Engel J, et al ILAE neuroimaging commission recommendations for neuroimaging of patients with epilepsy. Epilepsia. 1997;38:1–2.

[25] Brodie MJ, Dichter MA. Antiepileptic drugs. The New England journal of medicine. 1996;334(3):168–175. 8531974 10.1056/NEJM199601183340308

[26] Dichter MA, Brodie MJ. New antiepileptic drugs. The New England journal of medicine. 1996;334(24):1583–1590. 8628341 10.1056/NEJM199606133342407

[27] Sander JW. The use of antiepileptic drugs--principles and practice. Epilepsia. 2004;45 Suppl 6:28–34. 10.1111/j.0013-9580.2004.455005.x 15315513

[28] Perucca E, Dulac O, Shorvon S, Tomson T. Harnessing the clinical potential of antiepileptic drug therapy: dosage optimisation. Cns Drugs. 2001;15(8):609–621. 10.2165/00023210-200115080-00004 11524033

[29] Commission on Classification and Terminology of the International League Against Epilepsy. Proposal for revised clinical and electroencephalographic classification of epileptic seizures. Epilepsia. 1981;22(4):489–501. 6790275 10.1111/j.1528-1157.1981.tb06159.x

[30] Commission on Classification and Terminology of the International League Against Epilepsy.Proposal for revised classification of epilepsies and epileptic syndromes. Epilepsia. 1989;30(4):389–399. 2502382 10.1111/j.1528-1157.1989.tb05316.x

[31] Kwan P, Arzimanoglou A, Berg AT, Brodie MJ, Hauser WA, Mathern G, et al Definition of drug resistant epilepsy: Consensus proposal by the ad hoc Task Force of the ILAE Commission on Therapeutic Strategies. Epilepsia. 2010;51(6):1069–1077. 10.1111/j.1528-1167.2009.02397.x 19889013

[32] Patsalos PN, Berry DJ, Bourgeois BFD, Cloyd JC, Glauser TA, Johannessen SI, et al Antiepileptic drugs—best practice guidelines for therapeutic drug monitoring: A position paper by the subcommission on therapeutic drug monitoring, ILAE Commission on Therapeutic Strategies. Epilepsia. 2008;49(7):1239–1276. 10.1111/j.1528-1167.2008.01561.x 18397299

[33] Lhatoo SD, Wong IC, Polizzi G, Sander JW. Long-term retention rates of lamotrigine, gabapentin, and topiramate in chronic epilepsy. Epilepsia. 2000;41(12):1592–1596. 11114218 10.1111/j.1499-1654.2000.001592.x

[34] Dreyer NA, Garner S. Registries for Robust Evidence. Jama-J Am Med Assoc. 2009;302(7):790–791. 10.1001/jama.2009.1092 19690313

[35] Tomson T, Battino D, Craig J, Hernandez-Diaz S, Holmes LB, Lindhout D, et al Pregnancy registries: Differences, similarities, and possible harmonization. Epilepsia. 2010;51(5):909–915. 10.1111/j.1528-1167.2010.02525.x 20196792

[36] Vajda FJE, Hollingworth S, Graham J, Hitchcock AA, O'Brien TJ, Lander CM, et al Changing patterns of antiepileptic drug use in pregnant Australian women. Acta Neurol Scand. 2010;121(2):89–93. 10.1111/j.1600-0404.2009.01260.x WOS:000273395700003. 20015108

[37] Mohanraj R, Brodie MJ. Measuring the efficacy of antiepileptic drugs. Seizure: the journal of the British Epilepsy Association. 2003;12(7):413–443. 10.1016/s1059-1311(03)00047-5 12967570

[38] Shorvon S. Oxcarbazepine: a review. Seizure: the journal of the British Epilepsy Association. 2000;9(2):75–79. 10.1053/seiz.2000.0391 10845729

[39] Gilliam FG, Fessler AJ, Baker G, Vahle V, Carter J, Attarian H. Systematic screening allows reduction of adverse antiepileptic drug effects: a randomized trial. Neurology. 2004;62(1):23–27. 14718691 10.1212/wnl.62.1.23

[40] Hu Y, Lu Y, Yu W, Shen D, Xiao Z, Xi Z, et al Long-term retention rate of topiramate as initial monotherapy in Chinese patients with newly diagnosed epilepsy: A prospective, observational study. Epilepsy Res. 2010;90(3):278–284. 10.1016/j.eplepsyres.2010.06.004 20599359

[41] Tatum WO 4th, French JA, Faught E, Morris GL 3rd, Liporace J, Kanner A, et al Postmarketing experience with topiramate and cognition. Epilepsia. 2001;42(9):1134–1140. 11580760 10.1046/j.1528-1157.2001.41700.x

[42] Li J, Zheng R, Xu H, Fan T, Wang Y, Zhang X. A survey of Clinical application of Chinese patent antiepileptic medicine and pharmacy administration countermeasures in China.-author's translation. Chinese Journal of Pharmacoepidemiology. 2012;21(12):587–590. Epub 590.

[43] Cho Y-J, Heo K, Kim W-J, Jang SH, Jung YH, Ye BS, et al Long-term efficacy and tolerability of topiramate as add-on therapy in refractory partial epilepsy: An observational study. Epilepsia. 2009;50(8):1910–1919. 10.1111/j.1528-1167.2009.02177.x 19563348

[44] Hsieh FY, Lavori PW. Sample-size calculations for the Cox proportional hazards regression model with nonbinary covariates. Controlled clinical trials. 2000;21(6):552–560. 10.1016/s0197-2456(00)00104-5 11146149

