# Supplementary figures and images for: Comparative Long-Term Effectiveness of a Monotherapy with Five Antiepileptic Drugs for Focal Epilepsy in Adult Patients: A Prospective Cohort Study
Source: PLoS One. 2015 Jul 6;10(7):e0131566. doi: 10.1371/journal.pone.0131566 (PMC4493091; doi:10.1371/journal.pone.0131566)

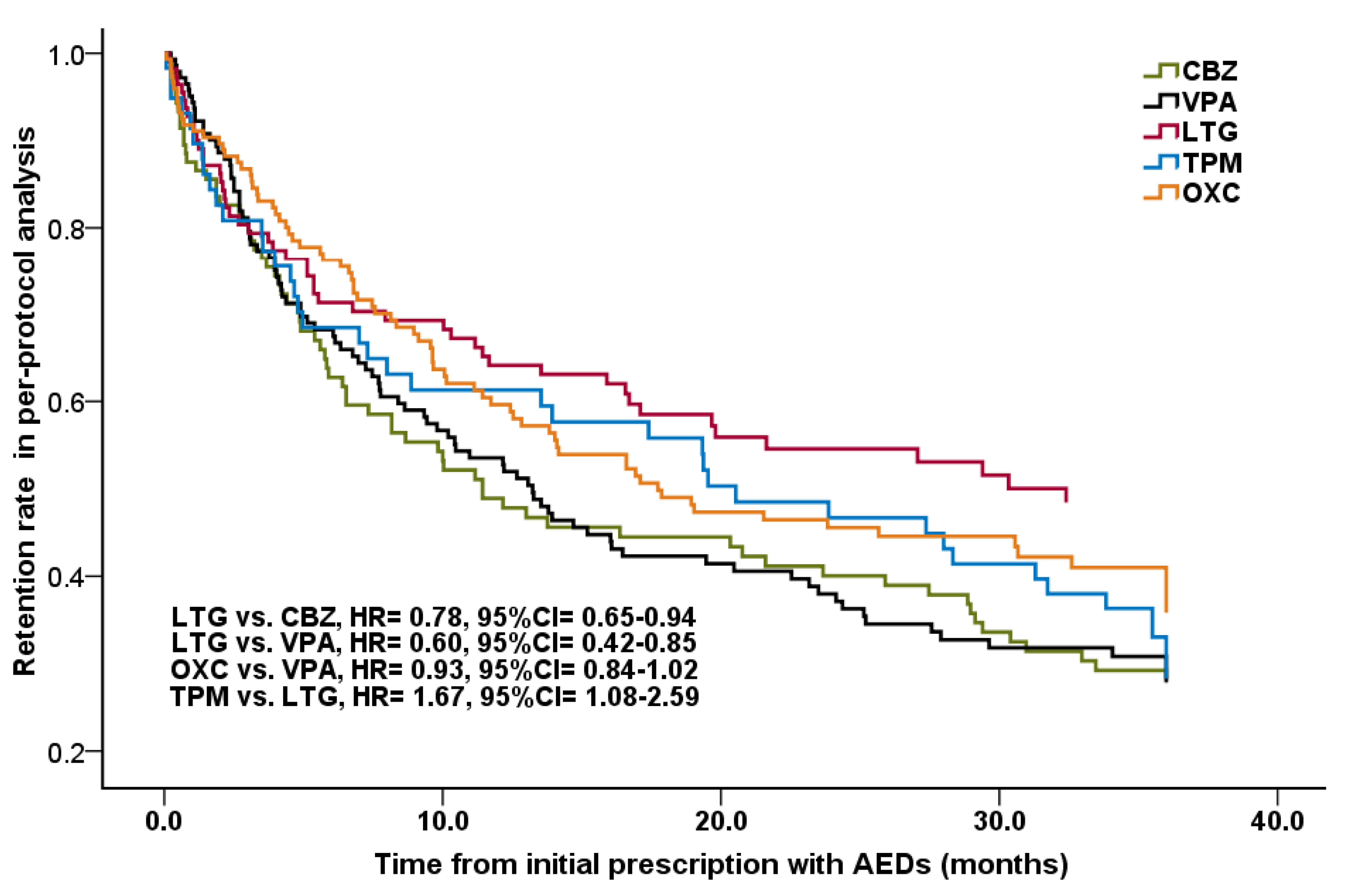

Supplement: S1 Fig — CBZ: carbamazepine; VPA: valproate; LTG: lamotrigine; TPM: topiramate; OXC: oxcarbazepine; AED: antiepileptic drug; HR: hazard ratio; CI: confidence interval. (TIF) [file pone.0131566.s001.tif]
