# Supplementary material for: Comparative Long-Term Effectiveness of a Monotherapy with Five Antiepileptic Drugs for Focal Epilepsy in Adult Patients: A Prospective Cohort Study
Source: PLoS One. 2015 Jul 6;10(7):e0131566. doi: 10.1371/journal.pone.0131566 (PMC4493091; doi:10.1371/journal.pone.0131566)
Supplement: S1 Table — AE: adverse event; compared with lamotrigine, *: P<0.05, **: P<0.01; compared with oxcarbazepine, #: P<0.05, ##: P<0.01; (DOCX) [file pone.0131566.s002.docx]

**S1 Table. Treatment failure rate of patients with AEs**

|  | **carbamazepine** | **valproate** | **lamotrigine** | **topiramate** | **oxcarbazepine** |
| --- | --- | --- | --- | --- | --- |
| Patients with at least one AE | 78 | 72 | 68 | 42 | 83 |
| Patients had treatment failure for AEs | 32 | 36 | 15 | 20 | 23 |
| Treatment failure rate of patients with AEs | 41.0%^*^ | 50.0%^**##^ | 22.1% | 47.6%^**#^ | 27.7% |

AE: adverse event; compared with lamotrigine, *: P<0.05, **: P<0.01; compared with oxcarbazepine, #: P<0.05, ##: P<0.01;
